# Evolutionary analysis of *Babesia vulpes* and *Babesia microti*-like parasites

**DOI:** 10.1186/s13071-022-05528-9

**Published:** 2022-11-03

**Authors:** Sanghyun Lee, Yeonchul Hong, Dong-Il Chung, Hyung-Kwan Jang, Youn-Kyoung Goo, Xuenan Xuan

**Affiliations:** 1 Division of Bio Bigdata, Department of Precision Medicine, Korea National Institute of Health, Korea Disease Control and Prevention Agency, Cheongju, Chungbuk South Korea; 2 Department of Parasitology and Tropical Medicine, School of Medicine, Kyungpook National University, Daegu, South Korea; 3 College of Veterinary Medicine, Jeonbuk National University, Iksan, Jeonbuk South Korea; 4 National Research Center for Protozoan Diseases, Obihiro University of Agriculture and Veterinary Medicine, Obihiro, Hokkaido Japan

**Keywords:** *Babesia vulpes*, South Korea, Raccoon dogs, *Babesia microti*-like parasite, Evolutionary analysis

## Abstract

**Background:**

The *Babesia microti-*like parasite is an emerging tick-borne piroplasm that has been detected in a range of hosts worldwide. *Babesia vulpes*, which is found in dogs and foxes, has been reclassified from *B. microti*-like parasites. The relationships among these *B. microti*-like parasites and *B. vulpes* with respect to host range and geographical origin have not been elucidated.

**Methods:**

Blood samples were collected from 27 raccoon dogs in South Korea and used to screen for *B. microti*-like parasites based on a PCR assay targeting the 18S rRNA gene of *Babesia*. For comparative purposes, in addition to 18S rRNA sequences from nine raccoon dogs, we also analyzed 18S rRNA sequences from *B. microti*-like parasites infecting hosts in different geographical regions worldwide obtained from the GenBank database, giving 123 sequences in total. The genetic variation and evolutionary relationships among these sequences were examined based on analyses using DnaSP, MEGA, Arlequine, and BEAST software.

**Results:**

*Babesia microti*-like parasites were identified in nine raccoon dogs and found to be related to *B. vulpes* obtained from Spanish dogs. Among the 123 sequences from 14 countries and various hosts, we identified 43 haplotypes with high genetic variance. Based on the genetic variance and phylogenetic analyses, we established that the *B. microti*-like parasites isolated in different geographical regions and from hosts belonging to five orders showed higher among-population variation than within-population variation. *Babesia vulpes* parasites infecting carnivore hosts, including raccoon dogs, foxes, skunks and dogs, appear to be genetically distinct from *B. microti*-like parasites infecting hosts belonging to the other orders.

**Conclusions:**

Our study demonstrated the genetic variation and evolutionary relationships among 18S rRNA sequences obtained from blood samples collected from various hosts and different geographical regions. *Babesia vulpes* was identified from raccoon dogs in South Korea. In addition, higher genetic variations were observed among populations of different hosts and geographical origins and, in particular, low connectivity was observed among host populations in the order Carnivora and those in other orders. These results suggest the *B. vulpes*, a piroplasmid species pathogenic in domestic dogs and wild canines, is genetically and evolutionarily different from *B. microti*-like parasites.

**Graphical Abstract:**

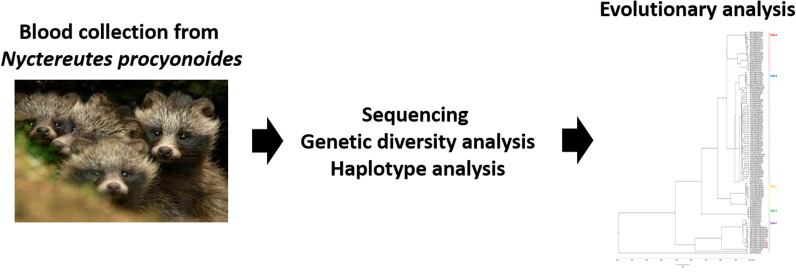

**Supplementary Information:**

The online version contains supplementary material available at 10.1186/s13071-022-05528-9.

## Background

Babesiosis, a disease transmitted by tick-borne piroplasms of the genus *Babesia*, is found in both wild and domestic animals and is receiving increasing attention as an emerging zoonotic disease [1]. One such parasite, *Babesia microti*, is considered to be a common parasite of rodents [2], although *B. microti*-like parasites have also been identified infecting a diverse range of host mammals, including carnivores, non-human primates and humans worldwide [3–7]. In humans, *B. microti* infection is characterized by fever and hemolysis, which are believed to be the primary signs of pathogenesis. Infections range from being asymptomatic to fulminant and can be complicated by respiratory failure, disseminated intravascular coagulation and/or organ failure [8]. To date, human cases of *B. microti*-like infection have been reported in China and Japan [9–11].

The *Babesia* genus was first discovered at the end of the nineteenth century, and subsequently many different species associated with domestic and wild animals have been described. Among the hosts belonging to the order Carnivora, *Babesia canis* and *Babesia gibsoni* were initially considered to be the two causal species associated with canine babesiosis, particularly in dogs [12]. However, *B. microti*-like parasites have been identified in a Spanish dog in Germany, and genetically similar *B. microti*-like parasites have been also identified in a range of hosts worldwide [13–16]. Baneth et al. reclassified this parasite as *Babesia vulpes*, which also has numerous synonyms, including *Babesia* sp. ‘Spanish dog,’ *B. microti*-like parasite and *Theileria annae* [17, 18]; to date, however, there is no consensus on the species name. In addition, parasites genetically similar to *B. microti* that have been identified in other hosts, including humans, are invariably referred to as *B. microti*-like parasites [19, 20]. In South Korea, *B. microti*-like parasites have been detected in asymptomatic raccoon dogs, water deer and Eurasian badgers [14, 21]. Consequently, given the general ambiguous nomenclature of these parasites, studies on the genetic variation and phylogenetics of *B. microti*-like parasites among populations from different geographical regions and hosts are warranted to clarify their taxonomy.

Accordingly, to gain further insights into the population structure of *B. microti*-like parasites in different hosts and geographical regions, we analyzed the genetic diversity and evolutionary characteristics of these parasites using 18S ribosomal RNA (18S rRNA) gene sequences collected from the GenBank database, along with those of isolates newly obtained from raccoon dogs (*Nyctereutes procyonoides*) in South Korea.

## Methods

### Sample collection and 18S rRNA gene amplification

Blood samples were collected from 27 raccoon dogs provided by the Jeonbuk National University in 2010 and 2011 (5 and 22 samples in 2010 and 2011, respectively). All samples were stored in ethylenediaminetetraacetic acid-containing tubes at − 70 °C until used for DNA extraction. Parasite genomic DNA was prepared from frozen blood stocks using QIAmp DNA Blood Kits (Qiagen, Hilden, Germany) following the manufacturer’s instructions. The extracted DNA was stored at − 70 °C until required.

Nested PCR was used to detect *Babesia* parasites in the extracted DNA samples obtained from raccoon dogs as previously described [19, 22]. The primary PCR amplification was conducted using the primer pair Bab1A (5′-GTCTTAGTATAAGCTTTTATACAGCG-3′) and Bab4A (5′-GATAGGTCAGAAACTTGAATGATACATCG-3′), followed by a second round of PCR amplification using 1 μl of the first-round PCR product as a template and the primer pair Bab2A (5′-CAGTTATAGTTTATTTGATGTTCGTTTTAC-3′) and Bab3A (5′-CGGCAAAGCCATGCGATTCGCTAAT-3′), in reaction mixtures containing 2.5 μl of 10× buffer, 2 μl of dNTPs, 1 μl each of forward and reverse primers, 0.25 μl of Ex Taq DNA Polymerase (Takara, Shiga, Japan) and distilled water to make up the volume to 25 μl. Electrophoresis was performed in 1.5% agarose gels containing 50 mg/ml ethidium bromide. In addition, the full-sized sequence (approx. 1.8 kb) encoding the 18S rRNA gene of the *Babesia* parasites was amplified in the DNA samples which tested positive for *Babesia* parasites, as previously described by Medlin et al. [23]. The 1.8-kb PCR products were then cloned and sequenced for use in genetic diversity and phylogenetic analyses. All 18S rRNA sequences obtained have been deposited in the GenBank database (Accession numbers: OM510434–OM510442).

### Analysis of the genetic diversity of* B. microti*-like parasites from South Korea

Initially, gene sequences were aligned using the default settings of the CLC Main Workbench 6 program (CLC Bio, Aarhus, Denmark), and the nucleotide composition, conserved sites, variable sites, parsimony informative sites and singleton sites were estimated using MEGA v.11 [24]. The program DnaSP v6 was employed to analyze the number of haplotypes [25]. Sequences showing even a single nucleotide difference were considered to be different haplotypes. Analysis of molecular variance (AMOVA) was performed using Arlequin v.3.5 [26] with 1000 non-parametric permutations (*P* = 0.05) to determine the proportions of genetic diversity within and among populations. To determine the levels of genetic differentiation among populations, for all datasets, we assessed pairwise fixation index (F_ST_) values between populations using Arlequin, with the significance of the evaluated F_ST_ values being based on 1000 random permutations. In addition, for assessing possible population expansion, we performed neutrality tests using Arlequin-implemented Tajima's D [27] and Fu's F_S_ [28].

### Comparative analysis of the genetic variability of worldwide isolates of* Babesia microti*-like parasites

To assess the worldwide genetic variability of *B. microti*-like parasites, we performed a comparative analysis of all *B. microti* 18S rRNA sequences available in the GenBank database (Additional file 1: Table S1). In addition, to estimate changes in population size over time, as well as the time to the most recent common ancestor (tMRCA) for each clade of *B. microti*-like parasites, we performed a Bayesian skyline plot analysis implemented in BEAST v2.5.0 [29], using a piecewise-constant skyline model. To reconstruct the demographic history over time, we used Tracer v1.5 [30], for which the best fitting nucleotide substitution model was selected using ModelFinder (ver. 2.0) based on the minimum Bayesian information criterion value. The appropriate tree was targeted using the TreeAnnotator included in the BEAST package by selecting the tree with the maximum sum of posterior probabilities (maximum clade credibility) after a 10% burn-in. Finally, the tree was visualized using FigTree (ver. 1.4).

## Results

### Identification of* B. vulpes* isolates from raccoon dogs in South Korea

**Fig. 1:**
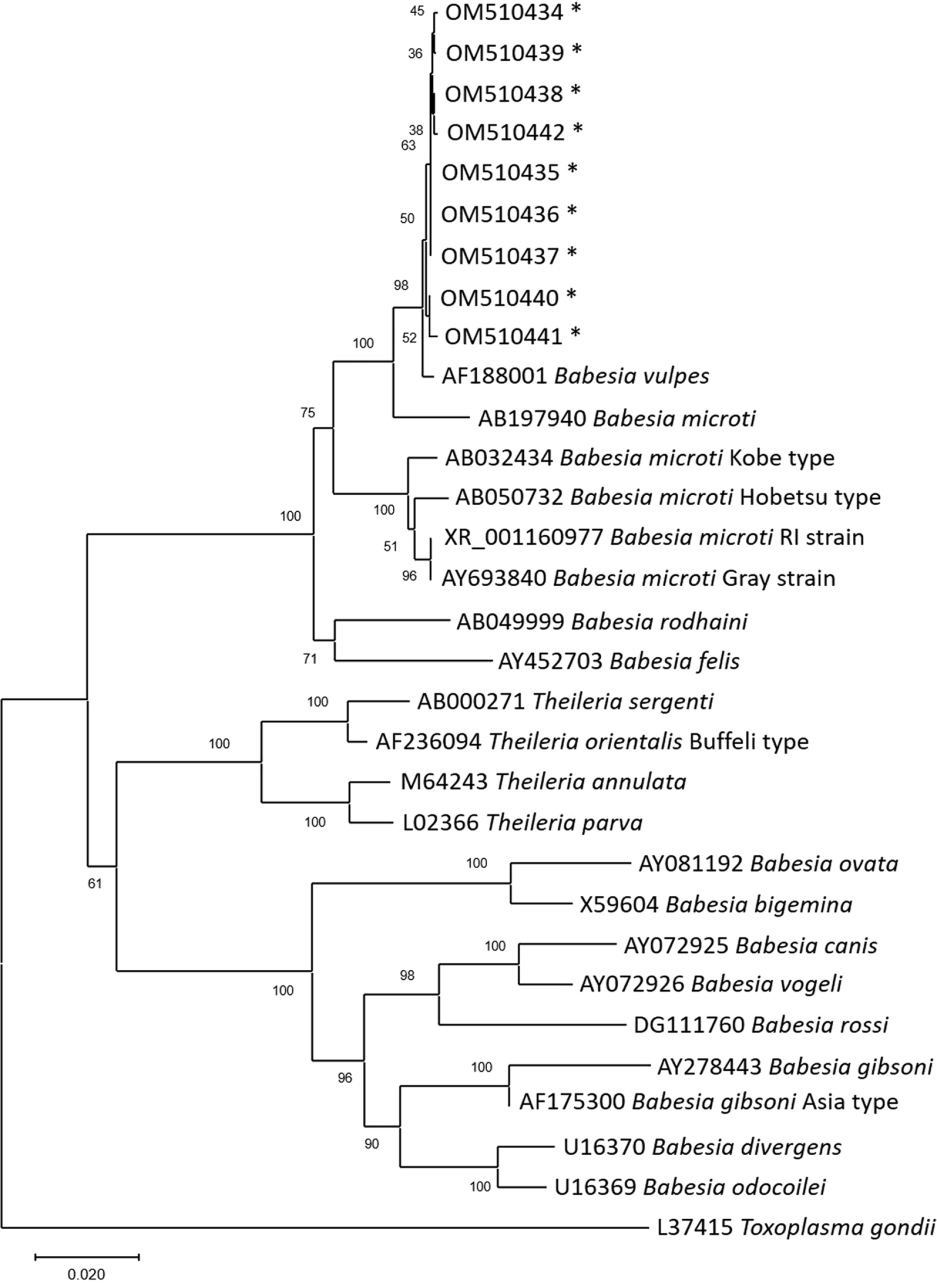
A neighbor-joining phylogenetic tree constructed based on the comparison of 18S ribosomal RNA gene sequences of *Babesia*-related parasites. *Toxoplasma gondii* was included as the outgroup. The numbers on each branch denote the percentage occurrence in 1000 bootstrap replicates. Asterisks indicate novel sequences identified in this study

**Fig. 2:**
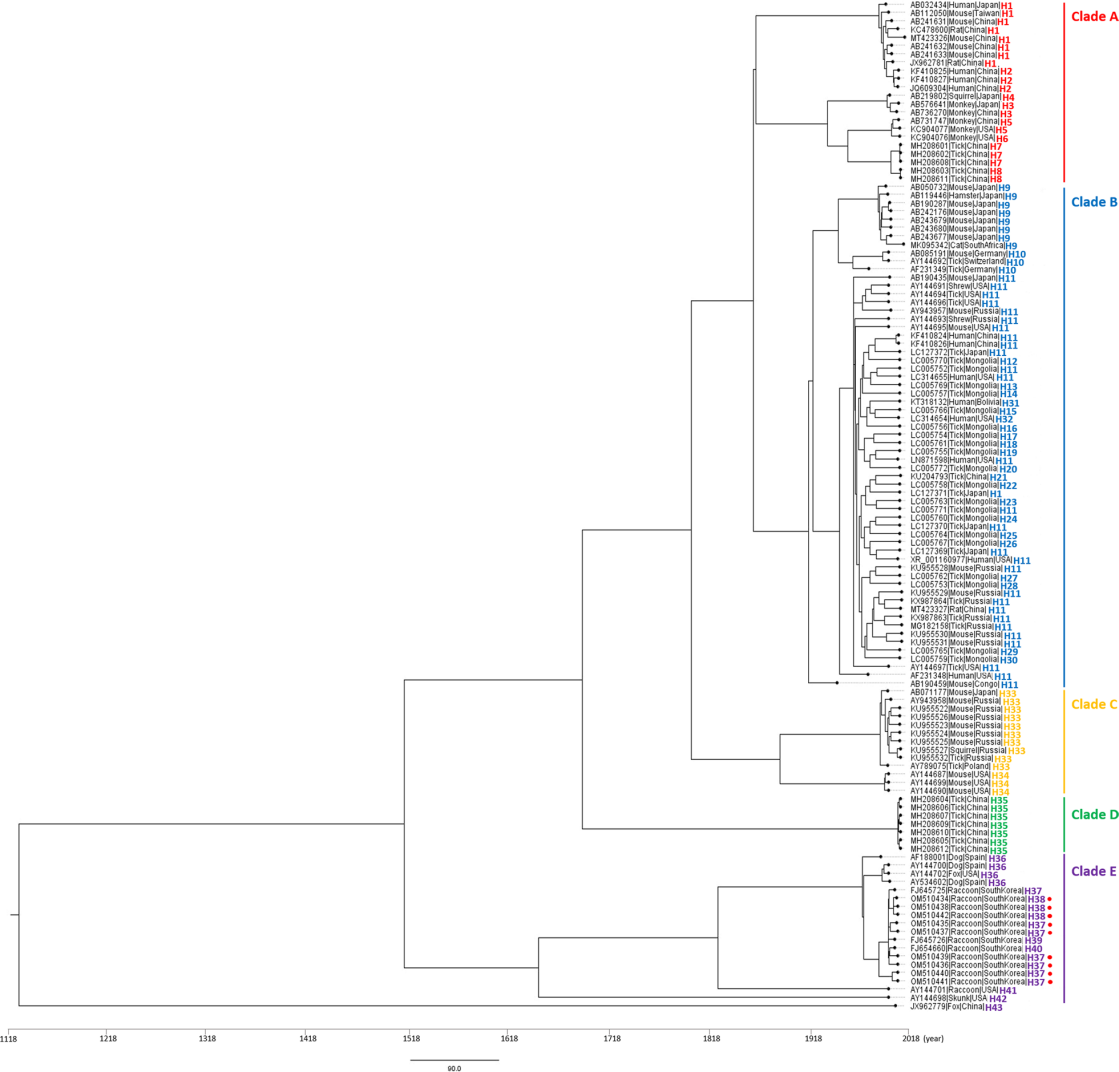
A phylogram of *Babesia microti*-like parasites and *B. vulpes* isolates obtained from hosts worldwide, with respective divergence time estimates. Red circles indicate novel sequences identified in this study. Haplotypes (H1—H43) of each isolate included are shown next to sequence name

Among the blood samples obtained from 27 raccoon dogs, nine samples (33.3%) showed evidence of *Babesia* infection. Sequencing of the nine amplicons revealed the presence of two different haplotypes (H37 and H38; Fig. 2; Additional file 1: Table S1), which showed 99.88% similarity. In addition, the isolates were found to have 99.58% similarity with the *B. microti* sequence deposited in GenBank by the Centers for Disease Control and Prevention (GenBank accession number: AY534602). The 18S rRNA gene sequences obtained in the present study have been deposited in GenBank under the accession numbers OM510434–OM510442. In the phylogenetic tree constructed for *Babesia* species and *Babesia*-related parasites, the identified sequences clustered with *B. microti* (Fig. 1). Specifically, we found that the parasites isolated from raccoon dogs clustered with *B. microti* parasites and with* Babesia* sp. isolated in Spanish dogs (previously referred as *B. vulpes*). Indeed, among carnivore hosts, only parasites that cause fulminating disease in Spanish dogs (*B. vulpes*) were included in same cluster as the parasites isolated from South Korean raccoon dogs. Based on these observations, we classified the isolates in this study as *B. vulpes*.

### Comparison of genetic variability among* B. vulpes* and* B. microti*-like parasites of different geographical origins

Analysis of the 123 sequences of the 18S rRNA gene from 14 countries indicated the presence of 43 haplotypes, whose alignment revealed nucleotide variations at 159 aligned positions (107 singleton variable sites and 52 parsimony informative sites) (Additional file 2: Figure S1). Pairwise comparisons among the 43 haplotypes revealed percentage identities ranging from 85.01% to 99.91% and nucleotide differences ranging from one to 162 (Additional file 3: Figure S2). Among the identified haplotypes, H11 was the most prominent, being detected in 29 sequences from four continents [Asia (China, Japan, Mongolia), Europe (Russia), North America (USA), Africa (Congo)]. Furthermore, 27 haplotypes (H4, H6, H12-H32, H39, H40, H42, H43) were identified as being unique to single samples, among which the H43 haplotype, detected in an isolate from a fox in China, was notably different from the other identified haplotypes.

In terms of geographical distribution, in Asia, haplotypes H2, H8, H35 were found in China, while H37, H38, H39 and H40 were specific to South Korea. Interestingly, sequences from Mongolia showed a high diversity, with 19 distinct haplotypes (H11-H20, H22-H30 and H32) being detected among the 20 sequences assessed. In North America, nine sequences were identified as the H11 haplotype, and H6, H31, H32, H34 and H42 were identified as unique haplotypes. In addition, haplotypes H10, H11, H33 and H36 were detected in Europe, and H11 and H9 were found in Africa. We also identified a number of haplotypes that were detected in more than one country. For example, in addition to the predominant H11 haplotype, haplotypes H9 and H33 detected in isolates from Japan were also identified among isolates from Russia and South Africa, respectively. Moreover, the three haplotypes H5, H36 and H41 identified among isolates from the USA were respectively detected in isolates from China, Spain and South Korea.

Analyses of genetic variance among and within populations based on AMOVA revealed that among all datasets, considerably more variance existed among the different geographical populations than within individual populations (60.12% vs 30.88%, respectively) (Table 1). As a measure of population differentiation, we determined the F_ST_ values between haplotypes from the four continents based on 18S rRNA sequences (Additional file 1: Table S2). Most of the F_ST_ values were close to 0, and apart from those between Europe and the remaining populations, none of the estimated pairwise F_ST_ values were statistically significant. In addition, we obtained significant negative neutrality test values for Tajima’s D and Fu’s F_S_ for the total population and Asian populations, with the exception of the significant positive value of Fu’s F_S_ (14.157, *P* < 0.02). Values obtained for the European and North American populations were positive but non-significant (Table 2).

**Table 1:** Analysis of molecular variance in populations of *Babesia vulpes* and *Babesia microti*-like parasites

Population definition	Source of variation							
	Among populations				Within populations			
	*df*	Sum of squares	Variance components	Percentage variation	*df*	Sum of squares	Variance components	Percentage variation
Host species	4	917.659	7.77678	62.1%	118	508.649	5.3973	21.0%
Geographical origin	3	136.159	1.14419	60.1%	119	64.718	0.86129	30.9%

*df* Degrees of freedom

**Table 2:** Results of neutrality tests for *B. vulpes* and *B. microti*-like populations in different geographical regions and host species

Population	Neutrality tests	
	Fu’s F_S_	Tajima’s D
G*eographical region*		
Asia	− 6.458^a^	− 2.14896*
Europe	14.157^a^	0.43602
North America	3.889	0.91540
Africa	NA	NA
*Host species*		
Primates	0.584	0.26485
Rodentia	7.438	0.14327
Carnivora	4.712*	2.23240*
Eulipotyphla	NA	NA
Ixodida	− 4.465*	− 1.46790*
All samples	− 8.451*	− 2.21984*

*NA *Not available

*Significant difference (*P* < 0.05)

### Comparison of the genetic variability among different hosts

In total, 123 sequences were obtained from samples from different host species belonging to five different orders, namely Primate, Rodentia, Carnivora, Eulipotyphla and Ixodida (Additional file 1: Table S1). Among the detected haplotypes, the predominant haplotype, H11, was identified in hosts belonging to four orders, the exception being Carnivora. In addition to H11, five haplotypes, namely H1, H3, H9, H10 and H33, were commonly identified among different hosts, whereas the remaining haplotypes were found in specific hosts. In the order Primate, nine different haplotypes, H1-H6, H11, H31 and H32, were detected in monkeys and humans, among which H2, H4, H5, H31 and H32 were unique to primates, whereas H1 and H3 were detected in hosts belonging to the order Rodentia. The 35 sequences obtained from rodents were characterized by the presence of the haplotypes H1, H3, H9-H11, H33 and H34 which, apart from H34, were also detected among the hosts belonging to other orders. Among the 21 sequences obtained from hosts belonging to the order Carnivora, we detected nine haplotypes (H9, H11, H37-H43) which, with the exception of the predominant H11 haplotype and H9 from a cat, were observed exclusively among carnivores. We also detected 25 different haplotypes among the 47 sequences obtained from hosts belonging to the order Ixodida, of which only three haplotypes, H10, H11 and H33, were also identified in the hosts belonging to other orders.

Similar to the results obtained for different geographical populations, for all datasets, we also detected considerably greater genetic variance among different host populations than within populations (62.05% vs 20.95%, respectively) (Table 1). Among the pairwise F_ST_ values obtained for comparisons between hosts belonging to the five assessed orders, higher values (> 0.5) were obtained for comparisons between carnivore hosts and those in other orders, which, with the exception of Eulipotyphla, were statistically significant (Additional file 1: Table S3). F_ST_ values obtained for other comparisons tended to be close to 0 with statistical significance, but they were non-significant for comparisons between hosts belonging to the order Eulipotyphla and those belonging to other orders. In addition, we obtained significant negative neutrality test Tajima’s D and Fu’s F_S_ values for the total population and Ixodida populations, whereas significant positive values were obtained for Carnivora populations (Table 2). Non-significant positive values were obtained for Primate and Rodentia populations, whereas isolates from Eulipotyphla were not analyzed owing to the very small sample size (*n* = 2).

### Phylogenetic diversity of *B. vulpes* and* B. microti*-like parasites

Phylogenetic analysis based on the 123 sequences of the 18S rRNA gene obtained from different hosts revealed that these isolates clustered into five discrete clades (Clades A–E) (Fig. 2). tMRCA of *B. microti*-like parasites was estimated to be 1126 years [95% bootstrap confidence interval (BCI): 1124–1128 year]. The H43 haplotype of an isolate obtained from a fox in China was evolutionarily quite different from that in isolates in other clades. Apart from H43, isolates containing the seven haplotypes H36–H42 comprised a single clade (Clade E for *B. vulpes*), whereas the isolates containing the remaining haplotypes were clustered among Clades A–D. The time of origin of Clade E, comprising isolates from hosts in the order Carnivora (dogs, foxes, raccoon dogs, skunks) in different countries is estimated to be approximately 1502 years (95% BCI: 1496–1509 years). Among the other clades, the earliest diversifications gave rise to Clade D (Chinese haplotype from ticks, H35), whereas Clade A contains *B. microti*-like isolates obtained from primates (humans and monkeys) and rodents (mice and rats) in Asian countries and the USA. In Clade C, *B. microti*-like parasites isolated from mice in Russia, Japan and the USA were genetically related to those isolated from squirrels and ticks in Russia and Poland. Clade B comprises isolates from different countries and hosts harboring a relatively large number of haplotypes (H9-H32); however, the sequences of those haplotypes (H9-H32) are very similar with more than 95.83% identity, as shown in Additional file 3: Fig. S2. In addition, the constructed phylogenetic tree tends to imply that Clade B isolates have evolved from a Congo mouse isolate.

## Discussion

Our examination of haplotypes in this study revealed that six and five of the 43 detected haplotypes were common in populations from different geographical regions and different hosts, respectively, thereby signifying that most of the identified haplotypes (> 86.0%) are unique to specific regions or hosts. Correspondingly, our AMOVA analyses revealed considerably greater genetic variation among populations than within populations (in terms of both geographical origins and hosts), thereby indicating that once having established infestation within a given area or host, *B. microti*-like parasites tend to evolve within the respective areas or hosts [31]. Furthermore, we detected a higher percentage of variation within populations of different geographical origin than in different hosts (Table 1). The higher between-population differentiation indicated by AMOVA analysis was confirmed by the low estimated pairwise F_ST_ values for populations inhabiting different geographical regions (Additional file 1: Table S2). Interestingly, compared with hosts in other orders, we obtained higher F_ST_ values for the populations belonging to the order Carnivora, thereby indicating that *B. vulpes* parasites infecting dogs, raccoon dogs, foxes and skunks have low connectivity with other host populations. In contrast, compared with other orders, we obtained lower F_ST_ values for host populations belonging to the order Ixodida, thereby implying that ticks function as vectors between host populations and would accordingly tend to have high population differentiation [32].

Phylogenetic analysis based on 123 sequences of the 18S rRNA gene of *B. vulpes* and *B. microti*-like parasites revealed that evolutionarily, Clade E for *B. vulpes*, comprising those sequences obtained from hosts belonging to the order Carnivora, branched at an early stage from isolates clustered in the remaining four clades (Clades A–D), which again tends to confirm that *B. vulpes* isolates infecting carnivore hosts are genetically different. On the other hand, *B. microti*-like parasites in Clades A–D comprised isolates obtained from different geographical regions and hosts, although those in Clades A and D were generally obtained from primates in the Asian countries (China and Japan) and Ixodida ticks in China, respectively. In addition, the *B. microti*-parasites in Clade B are likely to be evolutionarily related to each other in different hosts (Rodentia, Ixodida, Primates, Eulipotyphla) and geographic regions (Asia, Europe, Africa, North America). Although many haplotypes (Hap9-Hap32) were included in Clade B, the genetic similarity among the isolates was very high, as shown in Additional file 3: Figure S2. Collectively, our findings indicate that the *B. vulpes* parasites infecting hosts belonging to the order Carnivora are evolutionarily and genetically distinct from those infecting other hosts, and are typically characterized by low connectivity with other populations. Comparatively, the *B. microti*-like parasites infecting other hosts are probably more closely genetically related, albeit with between-population differentiation.

## Supplementary Information

**Additional file 1: Table S1.** Geographical and host origins of *Babesia microti*-like specimens studied herein. **Table S2.** Estimated pairwise F_ST_ values of sequences between different geographical populations of *B. microti*-like parasites. **Table S3.** Estimated pairwise F_ST_ values of sequences between different host populations of *B. microti*-like parasites.

**Additional file 2: Figure S1.** Alignment of the 43 haplotypes (Hap1-Hap43) representing *B. microti*-like parasites from North America, Africa, Asia and Europe. Identical bases are indicated by a dot. The number above each base indicates the alignment position of 18S rRNA.

**Additional file 3: Figure S2.** Pairwise comparison of sequence difference (number of nucleotides) and percenaget identity (%) among 43 18S rRNA haplotypes (Hap1-Hap43) representing *B. microti*-like parasites from North America, Africa, Asia and Europe.

## Data Availability

The data used to support the finding of this study are included within the article.

## Abbreviations

AMOVA: Analysis of molecular variance
BCI: Bootstrap confidence interval
F_ST_: Fixation index
tMRCA: Time to the most recent common ancestor
