# Supplementary material for: Evolutionary analysis of Babesia vulpes and Babesia microti-like parasites
Source: Parasit Vectors. 2022 Nov 3;15:404. doi: 10.1186/s13071-022-05528-9 (PMC9635067; doi:10.1186/s13071-022-05528-9)
Supplement: Supplementary file 1 — Additional file 1: Table S1. Geographical and host origins of Babesia microti-like specimens studied herein. Table S2. Estimated pairwise FST values of sequences between different geographical populations of B. microti-like parasites. Table S3. Estimated pairwise FST values of sequences between different host populations of B. microti-like parasites. [file 13071_2022_5528_MOESM1_ESM.docx]

Supplementary Table 1. Geographical and host origins of *Babesia microti*-like specimens studied herein.

| Geographical origin | | Host origin | | Sample size | Haplotypes** | Accession No. | References |
| --- | --- | --- | --- | --- | --- | --- | --- |
| Asia | China | Human | *Homo sapiens* | 5 | H2(3), H11(2) | KF410824, KF410825, KF410826,  KF410827, JQ609304, | Ruan et al., 2012 * Zhou et al., 2013 |
|  |  | Tick | *Ixodes sp.* | 13 | H7(3), H8(2),  H21, H35(7), | KU204793, MH208601, MH208602, MH208603,  MH208604, MH208605, MH208606, MH208607,  MH208608, MH208609, MH208610, MH208611,  MH208612 | Jiang and Jiang, 2015 * Li et al., 2018 * |
|  |  | Monkey | *Macaca mulatta* | 2 | H3, H5 | AB731747, AB736270 | Deng et al., 2012* |
|  |  | Mouse | *Niviventer confucianus* | 4 | H1(4) | AB241631, AB241632, AB241633,  MT423326 | Saito-Ito et al., 2008 Wei et al., 2020 |
|  |  | Rat | *Rattus losea* | 1 | H1 | JX962781 | Zhang and Jiang, 2012* |
|  |  |  | *Rattus norvegicus* | 2 | H1, H11 | KC478600, MT423327 | Zhao et al., 2013* Wei et al., 2020 |
|  |  | Fox | *Vulpes sp.* | 1 | H43 | JX962779 | Zhang and Jiang, 2012* |
|  | Japan | Mouse | *Apodemus sp.* | 5 | H9(4), H11 | AB050732, AB190435, AB243677,  AB243679, AB243680 | Wei et al., 2001 Saito-Ito et al., 2004* Saito-Ito et al., 2007 |
|  |  |  | *Mus musculus* | 1 | H33 | AB071177 | Tsuji et al., 2001* |
|  |  |  | *Myodes andersoni* | 2 | H9(2) | AB190287, AB242176 | Saito-Ito et al., 2004* Saito-Ito et al., 2007 |
|  |  | Tick | *Ixodes sp.* | 4 | H11(4) | LC127369, LC127370, LC127371, LC127372 | Zamoto-Niikura, et al., 2016 |
|  |  | Human | *Homo sapiens* | 1 | H1 | AB032434 | Saito-Ito et al., 2000 |
|  |  | Monkey | *Macaca fuscata* | 1 | H9 | AB576641 | Hirata et al., 2011 |
|  |  | Hamster | *Mesocricetus auratus* | 1 | H9 | AB119446 | Saito-Ito et al., 2004 |
|  |  | Raccoon | *Procyon lotor* | 1 | H16 | AB197940 | Kawabuchi et al., 2005. |
|  |  | Squirrel | *Sciurus vulgaris* | 1 | H3 | AB219802 | Tsuji et al., 2006 |
|  | Mongolia | Tick | *Ixodes sp.* | 20 | H11(2), H12, H13, H14,  H15, H16, H17, H18,  H19, H20, H22, H23  H24, H25, H26, H27,  H28, H29, H30, | LC005752, LC005753, LC005754, LC005755,  LC005756, LC005757, LC005758, LC005759,  LC005760, LC005761, LC005762, LC005763,  LC005764, LC005765, LC005766, LC005767,  LC005769, LC005770, LC005771, LC005772 | Yokoyama et al., 2014* |
|  | South Korea | Raccoon Dog | *Nyctereutes procyonoides* | 12 | H37(7), H38(3), H39, H40 | FJ645725, FJ645726, FJ654660, OM510434,  OM510435, OM510436, OM510437, OM510438,  OM510439, OM510440, OM510441, OM510442 | Han and Na. 2009* Current study |
|  |  | Eurasian Badger | *Meles meles* | 1 | H35 | No accession number | Hong et al., 2017 |
|  | Taiwan | Rat | Rattus coxinga | 1 | H1 | AB112050 | Saito-Ito et al., 2003* |

*, Sequences were directly submitted to GenBank

**, The numbers in parentheses refer to the number of the sequence, and the number is omitted for a single sequence.

Supplementary Table 1. Geographical and host origins of *Babesia microti*-like specimens studied herein (continued).

| Geographical origin | | Host origin |  | Sample size | Haplotypes** | Accession No. | References |
| --- | --- | --- | --- | --- | --- | --- | --- |
| Europe | Germany | Mouse | *Clethrionomys glareolus* | 1 | H10 | AB085191 | Tsuji et al., 2002* |
|  |  | Tick | *Ixodes sp.* | 1 | H10 | AF231349 | Zahler et al., 2000 |
|  | Poland | Tick | Ixodes sp. | 1 | H33 | AY789075 | Pieniazek et al., 2006 |
|  | Russia | Mouse | *Clethrionomys rutilus* | 1 | H33 | AY943958 | Rar et al., 2011 |
|  |  |  | *Microtus sp.* | 4 | H11(2), H33(2) | AY943957, KU955531, KU955523,  KU955525 | Rar et al., 2011 Rar et al., 2016 |
|  |  |  | *Myodes sp.* | 6 | H11(3), H33(3) | KU955522, KU955524, KU955526,  KU955528, KU955529, KU955530 | Rar et al., 2016 |
|  |  | Eurasian shrew | *Sorex araneus* | 1 | H11 | AY144693 | Goethert et al., 2002* |
|  |  | Tick | *Ixodes sp.* | 4 | H11(3), H33 | MG182158, KU955532, KX987863,  KX987864 | Rar et al., 2016 |
|  |  | Squirrel | *Tamias sibiricus* | 1 | H33 | KU955527 | Rar et al., 2016 |
|  | Spain | Dog | *Canis lupus familiaris* | 3 | H36(3), | AF188001, AY144700, AY534602 | Zahler et al., 2000 Goethert et al., 2002* Slemenda et al., 2004* |
|  | Switzerland | Tick | Ixodes sp. | 1 | H10 | AY144692 | Goethert and Telford, 2002* |
| America | Bolivia | Human | Homo sapiens | 1 | H31 | KT318132 | Gabrielli, 2015* |
|  | United States of America | Human | *Homo sapiens* | 4 | H11(3), H32 | AF231348, LC314654, LC314655,  XR_001160977 | Zahler et al., 2000 Cornillot et al., 2012, 2013;  Silva et al., 2016.  Sayama et al., 2018 |
|  |  | Mouse | *Clethrionomys rutilus* | 2 | H34(2) | AY144687, AY144690 | Goethert et al., 2003 |
|  |  |  | *Microtus sp.* | 1 | H34 | AY144699 | Goethert and Telford, 2002* |
|  |  |  | *Peromyscus sp.* | 1 | H11 | AY144695 | Goethert and Telford, 2002* |
|  |  | Eurasian shrew | *Sorex araneus* | 1 | H11 | AY144691 | Goethert et al., 2003 |

*, Sequences were directly submitted to GenBank

**, The numbers in parentheses refer to the number of the sequence, and the number is omitted for a single sequence.

Supplementary Table 1. Geographical and host origins of *Babesia microti*-like specimens studied herein (continued).

| Geographical origin | | Host origin |  | Sample size | Haplotypes** | Accession No. | References |
| --- | --- | --- | --- | --- | --- | --- | --- |
| America | United States of America | Tick | *Ixodes* sp. | 3 | H11(3) | AY144694, AY144696, AY144697 | Goethert and Telford, 2002* |
|  |  | Skunk | *Mephitidae* | 1 | H42 | AY144698 | Goethert and Telford, 2002* |
|  |  | Monkey | *Rhesus macaque* | 2 | H5, H6 | KC904076, KC904077 | Liu et al., 2014 |
|  |  | Raccoon | *Procyon lotor* | 1 | H41 | AY144701 | Goethert and Telford, 2002* |
|  |  | Fox | *Vulpes* sp | 1 | H36 | AY144702 | Goethert and Telford, 2002* |
| Africa | Congo | Hamster | *Mesocricetus auratus* | 1 | H11 | AB190459 | Saito-Ito et al., 2004* |
|  | South Africa | Cat | *Felis catus* | 1 | H9 | MK095342 | Bosman et al., 2019 |

*, Sequences were directly submitted to GenBank

**, The numbers in parentheses refer to the number of the sequence, and the number is omitted for a single sequence.

**Supplementary Table 2.** Estimated pairwise *F*_ST_ values of sequences between different geographical populations of *Babesia microti*-like parasites.

|  | Asia | America | Europe | Africa |
| --- | --- | --- | --- | --- |
| Asia | 0.0000 |  |  |  |
| America | 0.0256 | 0.0000 |  |  |
| Europe | 0.0583* | 0.0637* | 0.0000 |  |
| Africa | 0.0002 | 0.0125 | 0.0558 | 0.0000 |

**Supplementary Table 3.** Estimated pairwise *F*_ST_ values of sequences between different host populations of *Babesia microti*-like parasites.

|  | Primate | Rodentia | Eulipotyphla | Carnivora | Ixodida |
| --- | --- | --- | --- | --- | --- |
| Primate | 0.0000 |  |  |  |  |
| Rodentia | 0.0942* | 0.000 |  |  |  |
| Eulipotyphla | 0.1017 | 0.0513 | 0.000 |  |  |
| Carnivora | 0.5560* | 0.5543* | 0.4900 | 0.000 |  |
| Ixodida | 0.0984* | 0.1050* | 0.1862 | 0.6152* | 0.000 |

**Supplementary references**

Bosman AM, Penzhorn BL, Brayton KA, Schoeman T, Oosthuizen MC. A novel *Babesia* sp. associated with clinical signs of babesiosis in domestic cats in South Africa. Parasit Vectors. 2019;12:138.

Cornillot E, Dassouli A, Garg A, Pachikara N, Randazzo S, Depoix D, et al. Whole genome mapping and re-organization of the nuclear and mitochondrial genomes of *Babesia microti* isolates. PLoS One. 2013;8:e72657.

Cornillot E, Hadj-Kaddour K, Dassouli A, Noel B, Ranwez V, Vacherie B, et al. Sequencing of the smallest Apicomplexan genome from the human pathogen *Babesia microti*. Nucleic Acids Res. 2012;40:9102-14.

Deng W, Hirata H, Wei Q, Qin C, Ishihara C. Identification and phylogenetic analysis of Chinese Macaque *Babesia* detected from a chinese macaque. Unpublished. Direct submission to GenBank. 2012.

Gabrielli S. Unpublished. Direct Submission to GenBank. 2015.

Goethert HK, Lubelcyzk C, LaCombe E, Holman M, Rand P, Smith RP Jr, et al. Enzootic *Babesia microti* in Maine. J Parasitol. 2003;89:1069-71.

Goethert H, Telford S III. What is *Babesia microti*? A phylogenetic analysis. Unpublished. Direct Submission to GenBank. 2002.

Han JI, Na KJ. Theileriosis of wild raccoon dogs in South Korea. Unpublished. Direct Submission to GenBank. 2009.

Hirata H, Kawai S, Maeda M, Jinnai M, Fujisawa K, Katakai Y, et al. Identification and phylogenetic analysis of Japanese Macaque *Babesia*-1 (JM-1) detected from a Japanese Macaque (Macaca fuscata fuscata). Am J Trop Med Hyg. 2011;85:635-8.

Jiang R, Jiang J. Genetic Diversity and Coexistence of *Babesia* in Ticks (Acari: Ixodidae) from Northeastern China. Unpublished. Direct Submission to GenBank. 2015.

Hong SH, Kim HJ, Jeong YI, Cho SH, Lee WJ, Kim JT, et al. Serological and Molecular Detection of *Toxoplasma gondii* and *Babesia microti* in the Blood of Rescued Wild Animals in Gangwon-do (Province), Korea. Korean J Parasitol. 2017;55:207-212.

Kawabuchi T, Tsuji M, Sado A, Matoba Y, Asakawa M, Ishihara C. *Babesia microti*-like parasites detected in feral raccoons (*Procyon lotor*) captured in Hokkaido, Japan. J Vet Med Sci. 2005;67:825-7.

Liu DX, Gill A, Holman PJ, Didier PJ, Blanchard JL, Veazey RS, Lackner AA. Persistent babesiosis in a Rhesus macaque (*Macaca mulatta*) infected with a simian-human immunodeficiency virus. J Med Primatol. 2014;43:206-8.

Li LH, Zhou XN, Zhang Y, Zhu D. Molecular survey on ticks and piroplasmida in Tengchong, Yunnan, China. Unpublished. Direct Submission to GenBank. 2018.

Pieniazek N, Sawczuk M, Skotarczak B. Molecular identification of *Babesia* parasites isolated from *Ixodes ricinus* ticks collected in northwestern Poland. J Parasitol. 2006;92:32-5.

Rar VA, Epikhina TI, Livanova NN, Panov VV. Genetic diversity of *Babesia* in *Ixodes persulcatus* and small mammals from North Ural and West Siberia, Russia. Parasitology. 2011;138:175-82.

Rar V, Yakimenko V, Makenov M, Tikunov A, Epikhina T, Tancev A, et al. High prevalence of *Babesia microti* 'Munich' type in small mammals from an *Ixodes persulcatus*/*Ixodes trianguliceps* sympatric area in the Omsk region, Russia. Parasitol Res. 2016;115:3619-29.

Ruan W, Yao L, Zhang X, Lu, Q. The molecular identification of a human *Babesia* and analysis of its sequence. Unpublished. Direct Submission to GenBank. 2012.

Saito-Ito A, Dantrakool A, Takada N, Chen ER. Comparison between *Babesia microti*-like parasites in Taiwan and Japan, two human babesiosis-emergence countries in Asia. Unpublished. Direct Submission to GenBank. 2004.

Saito-Ito A, Dantrakool A, Yano Y, Takada N, Chen E. Descrimination between *Babesia microti* isolates with the same sequence of small subunit ribosomal RNA gene in Taiwan and Japan by the sequence of internal transcribed spacers. Unpublished. Direct Submission to GenBank. 2003.

Saito-Ito A, Kasahara M, Kasai M, Dantrakool A, Kawai A, Fujita H, et al. Survey of *Babesia microti* infection in field rodents in Japan: records of the Kobe-type in new foci and findings of a new type related to the Otsu-type. Microbiol Immunol. 2007;51:15-24.

Saito-Ito A, Takada N, Ishiguro F, Fujita H, Yano Y, Ma XH, et al. Detection of Kobe-type *Babesia microti* associated with Japanese human babesiosis in field rodents in central Taiwan and southeastern mainland China. Parasitology. 2008;135:691-9.

Saito-Ito A, Tsuji M, Wei Q, He S, Matsui T, Kohsaki M, et al. Transfusion-acquired, autochthonous human babesiosis in Japan: isolation of *Babesia microti*-like parasites with hu-RBC-SCID mice. J Clin Microbiol. 2000;38:4511-6.

Saito-Ito A, Yano Y, Dantrakool A, Hashimoto T, Takada N. Survey of rodents and ticks in human babesiosis emergence area in Japan: first detection of *Babesia microti*-like parasites in *Ixodes ovatus*. J Clin Microbiol. 2004;42:2268-70.

Sayama Y, Zamoto-Niikura A, Matsumoto C, Saijo M, Ishihara C, Matsubayashi K, et al. Analysis of antigen-antibody cross-reactivity among lineages and sublineages of *Babesia microti* parasites using human babesiosis specimens. Transfusion. 2018;58:1234-1244.

Silva JC, Cornillot E, McCracken C, Usmani-Brown S, Dwivedi A, Ifeonu OO, et al. Genome-wide diversity and gene expression profiling of *Babesia microti* isolates identify polymorphic genes that mediate host-pathogen interactions. Sci Rep. 2016;6:35284.

Slemenda SB, Camacho AT, Guitian FJ, Pallas E, Gestal JJ, Olmeda AS, et al. Infections of dogs in northwest Spain with a *Babesia microti*-like organism. Unpublished. Direct Submission to GenBank. 2004.

Tsuji M. Phylogenetic analysis of *Babesia microti*-like parasites. Unpublished. Direct Submission to GenBank. 2001.

Tsuji M, Zamoto A, Gray J. Phylogenetic analysis for *Babesia microti* isolated in Europe. Unpublished. Direct Submission to GenBank. 2002.

Tsuji M, Zamoto A, Kawabuchi T, Kataoka T, Nakajima R, Asakawa M, et al. *Babesia microti*-like parasites detected in Eurasian red squirrels (*Sciurus vulgaris* orientis) in Hokkaido, Japan. J Vet Med Sci. 2006;68:643-6.

Wei CY, Wang XM, Wang ZS, Wang ZH, Guan ZZ, Zhang LH, Dou XF, Wang H. High prevalence of *Babesia microti* in small mammals in Beijing. Infect Dis Poverty. 2020 Nov 11;9(1):155. doi: 10.1186/s40249-020-00775-3. PMID: 33176879; PMCID: PMC7661193.

Wei Q, Tsuji M, Zamoto A, Kohsaki M, Matsui T, Shiota T, Telford SR 3rd, Ishihara C. Human babesiosis in Japan: isolation of *Babesia microti*-like parasites from an asymptomatic transfusion donor and from a rodent from an area where babesiosis is endemic. J Clin Microbiol. 2001 Jun;39(6):2178-83. doi: 10.1128/JCM.39.6.2178-2183.2001. PMID: 11376054; PMCID: PMC88108.

Yokoyama N. Babesia microti in questing ticks in Mongolia. Unpublished. Direct Submission to GenBank. 2014.

Zahler M, Rinder H, Gothe R. Genotypic status of *Babesia microti* within the piroplasms. Parasitol Res. 2000;86:642-6.

Zamoto-Niikura A, Morikawa S, Hanaki KI, Holman PJ, Ishihara C. Ixodes persulcatus Ticks as Vectors for the *Babesia microti* U.S. Lineage in Japan. Appl Environ Microbiol. 2016 Oct 27;82(22):6624-6632. doi: 10.1128/AEM.02373-16. PMID: 27590815; PMCID: PMC5086556.

Zhang Y, Jiang JF. Unpublished. Direct Submission to GenBank. 2012.

Zhao X, Li H, Liu W, Cao W. Coinfection of *Anaplasma phagocytophilum* and *Babesia microti* in rodents in China. Unpublished. Direct Submission to GenBank. 2013.

Zhou X, Li SG, Chen SB, Wang JZ, Xu B, Zhou HJ, et al. Co-infections with *Babesia microti* and *Plasmodium* parasites along the China-Myanmar border. Infect Dis Poverty. 2013;2:24.
