# Supplementary material for: Evolutionary analysis of Babesia vulpes and Babesia microti-like parasites
Source: Parasit Vectors. 2022 Nov 3;15:404. doi: 10.1186/s13071-022-05528-9 (PMC9635067; doi:10.1186/s13071-022-05528-9)
Supplement: Supplementary file 2 — Additional file 2: Figure S1. Alignment of the 43 haplotypes (Hap1-Hap43) representing B. microti-like parasites from North America, Africa, Asia and Europe. Identical bases are indicated by a dot. The number above each base indicates the alignment position of 18S rRNA. [file 13071_2022_5528_MOESM2_ESM.docx]

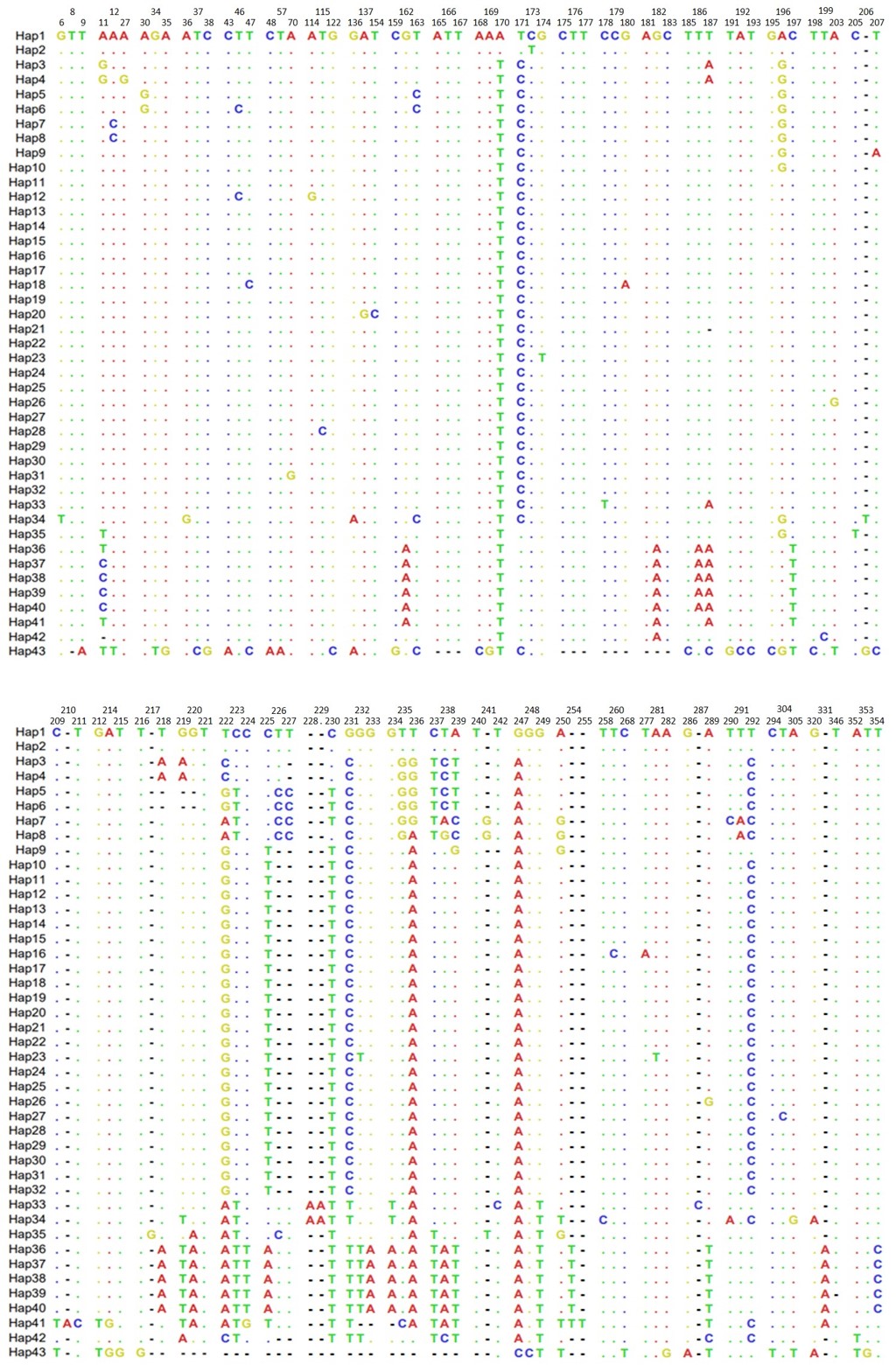


**Supplementary Figure 1.** Alignment of the 43 haplotypes (Hap1-Hap43) representing *Babesia microti*-like parasites from America, Africa, Asia, and Europe. Identical bases are indicated by a dot. The number above each base indicates the alignment position of 18S rRNA.


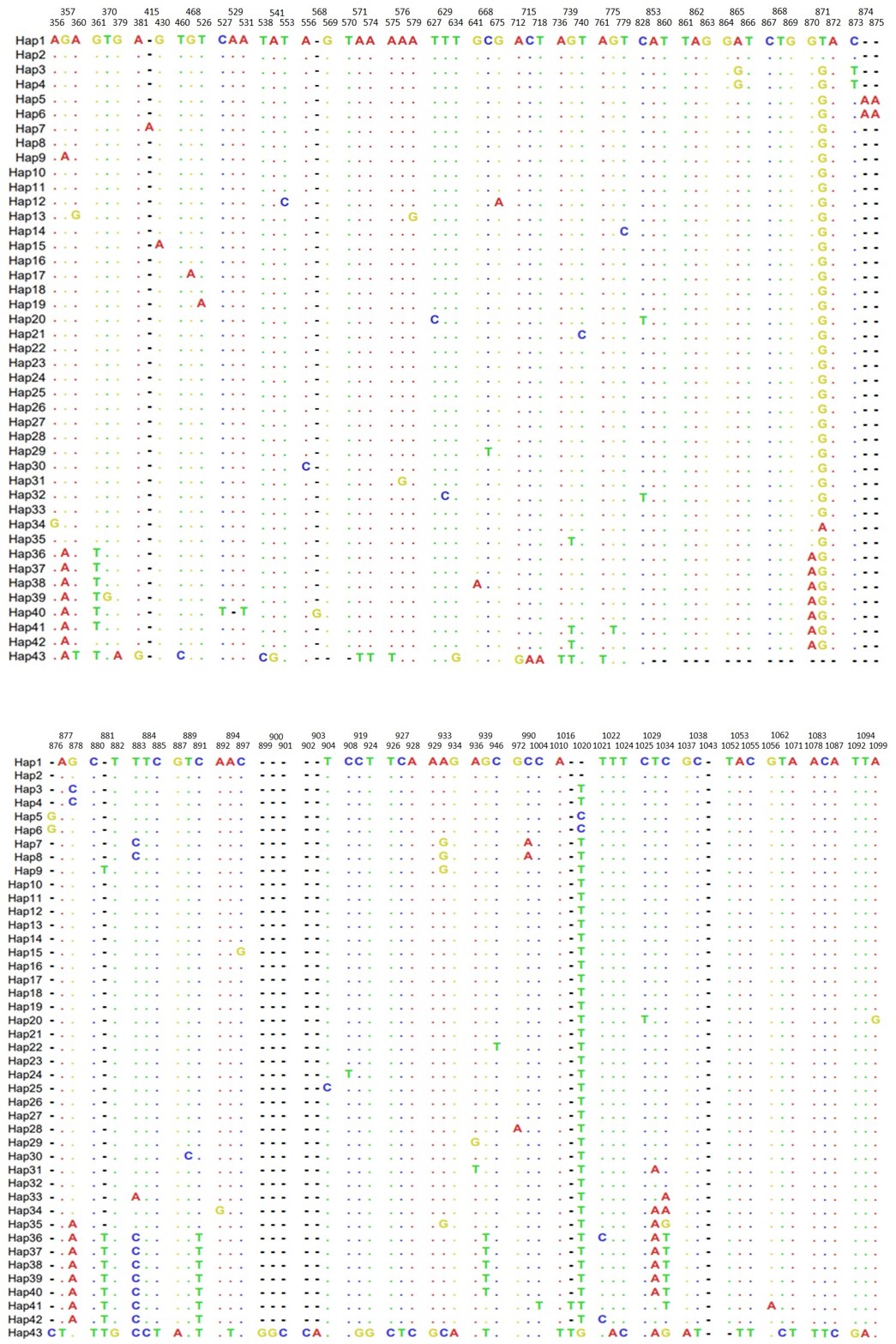


**Supplementary Figure 1 (continued).** Alignment of the 43 haplotypes (Hap1-Hap43) representing *Babesia microti*-like parasites from America, Africa, Asia, and Europe. Identical bases are indicated by a dot. The number above each base indicates the alignment position of 18S rRNA.
