# Supplementary material for: Evolutionary analysis of Babesia vulpes and Babesia microti-like parasites
Source: Parasit Vectors. 2022 Nov 3;15:404. doi: 10.1186/s13071-022-05528-9 (PMC9635067; doi:10.1186/s13071-022-05528-9)
Supplement: Supplementary file 3 — Additional file 3: Figure S2. Pairwise comparison of sequence difference (number of nucleotides) and percenaget identity (%) among 43 18S rRNA haplotypes (Hap1-Hap43) representing B. microti-like parasites from North America, Africa, Asia and Europe. [file 13071_2022_5528_MOESM3_ESM.docx]

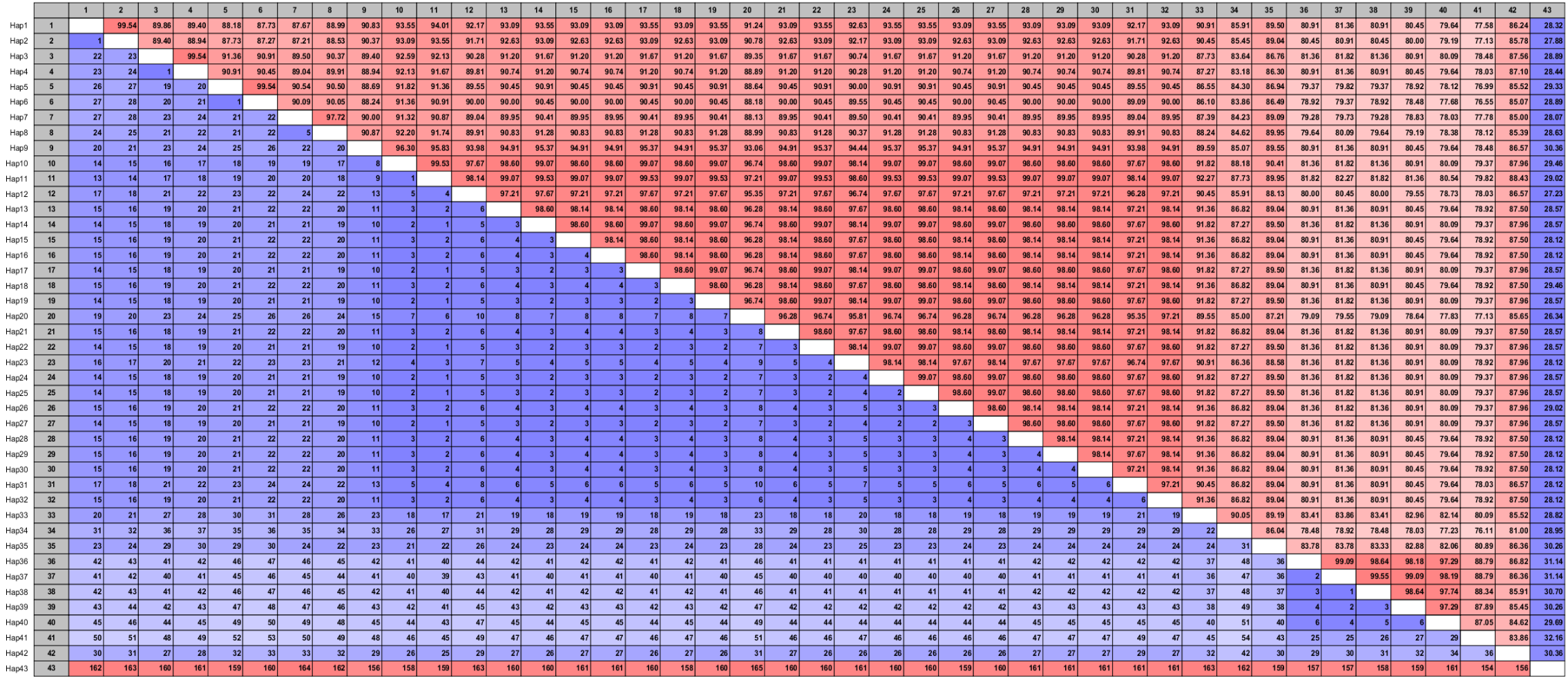


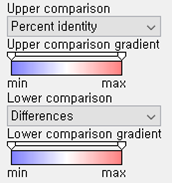


**Supplementary Figure 2.** Pairwise comparison of sequence difference (number of nucleotides) and percent identity (%) among 4318S rRNA haplotypes (Hap1-Hap43) representing *Babesia microti*-like parasites from America, Africa, Asia, and Europe.
